# Adsorption of Ciprofloxacin on Clay Minerals in Argentinian Santa Rosa-Corrientes Soils

**DOI:** 10.3390/molecules29081760

**Published:** 2024-04-12

**Authors:** Nelly L. Jorge, María V. Garrafa, Jorge M. Romero, María J. Jorge, Lilian C. Jorge, Mario R. Delfino, Yumeida V. Meruvia-Rojas, Alfonso Hernández-Laguna, C. Ignacio Sainz-Díaz

**Affiliations:** 1 Laboratorio de Investigaciones en Tecnología del Medio Ambiente, Área de Química Física, Facultad de Ciencias Exactas y Naturales y Agrimensura, Universidad del Nordeste, Corrientes 3400, Argentina; lidianj@exa.unne.edu.ar (N.L.J.); mavigarrafa@gmail.com (M.V.G.); jromero@exa.unne.edu.ar (J.M.R.); maria.jorge@comunidad.unne.edu.ar (M.J.J.); liliancj@vet.unne.edu.ar (L.C.J.); 2 Instrumental Analysis Laboratory, Facultad de Ciencias Exactas y Naturales y Agrimensura, Universidad del Nordeste, Av. Libertad 5460, Corrientes 3440, Argentina; mariodelfino@exa.unne.edu.ar; 3 Instituto Andaluz de Ciencias de la Tierra (CSIC-UGR), Av. de las Palmeras 4, 18100 Armilla, Granada, Spain; yumeidameros@gmail.com

**Keywords:** montmorillonite, ciprofloxacin, soil pollution, adsorption/desorption isotherms, DFT, force field, molecular modelling

## Abstract

The presence of antibiotics in soils is increasing drastically in last decades due to the intensive farming industry and excessive human consumption. Clay minerals are one of the soil components with great adsorption capacity for organic pollutants. The study of interactions between antibiotics and mineral surfaces will give us scientific knowledge of these pollutants through soils. In this work, we study the adsorption of the antibiotic ciprofloxacin in the clay mineral fraction of soils from the Argentinian zone of Santa Rosa (Corrientes), in a collaborative research of experiments and atomistic modelling calculations of the intercalation of ciprofloxacin in the interlayer space of montmorillonite. Adsorption and desorption isotherms were performed and compared with different isotherm models. Additionally, enthalpy, entropy, and free energy were determined from equilibrium constants at a function of temperature. All these experiments and calculations lead to the conclusions that two adsorption types of ciprofloxacin are found on clay minerals: one weakly sorbed that is released during the desorption experiments, and other one strongly joined that remains in the soil.

## 1. Introduction

The pollution of soils by antibiotics is becoming a serious environmental issue. Intensive farming activities enhance antibiotic abuse for increasing productivity and preventing animal diseases. This use is also extended to fish–livestock factories. Consequently, the manure and wastewater generated by these industries can contaminate soils with antimicrobials all around the world [1,2,3,4]. For example, a high concentration of antibiotics was detected in the Chinese Pearl River [5]. The worldwide use of antibiotics is mainly (73%) in livestock farms [6].

In Argentina, various agricultural activities, mainly livestock and poultry farming, use a wide range of pharmaceutical products (anthelmintics, hormones, and antibiotics, among others) for therapeutic and non-therapeutic purposes, to promote animal health and increase productivity. Some of these drugs are administered to the entire herd for the prevention of infectious diseases, a process called metaphylaxis. Both the intensive raising of cattle and hens produce large quantities of waste in the form of manure, urine, guano, chicken litter, as well as different effluents. Then, part of the non-metabolized antibiotics reach the environment [7].

Recent studies in Argentina have reported the presence of antibiotics for veterinary use in surface waters in many provinces [7]. In Corrientes, there are many farming activities, and manure and chicken litter are used as fertilizers. However, there is not much work on how these practices affect the environment in this region. For this reason, our study is focused on one of these antibiotics (ciprofloxacin) for observing if correct doses are used and monitoring the waste management process.

From these contaminations, these waste antibiotics can reach the human food chain producing health problems, such as allergies, resistance, and other possible diseases. This issue is worsened by the slow degradation and persistence in the environment of these pollutants [8,9,10]. The knowledge of the accumulation and diffusion mechanisms of these antibiotics through soils is necessary for evaluating the pollution and health risks.

**Figure 1:**
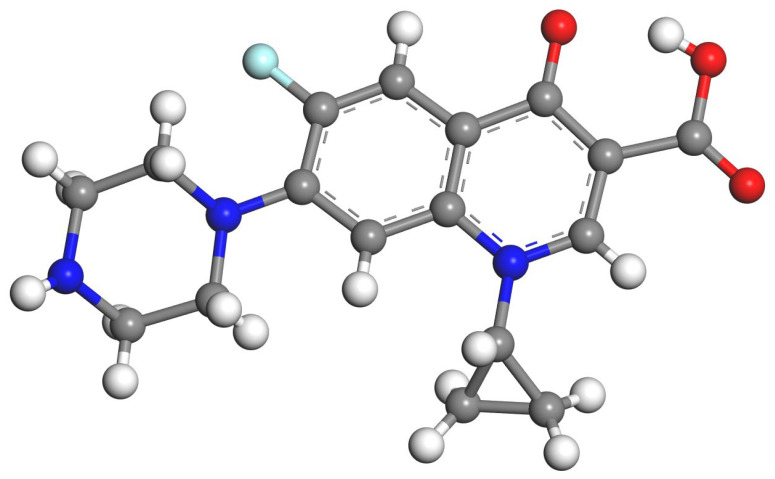
Molecular structure of ciprofloxacin. The H, C, O, F, and N atoms are in white, grey, red, clear-blue, and blue, respectively. This color convention is applied to the rest of this work.

Ciprofloxacin (cipro) [1-cyclopropyl-6-flouro-1,4-dihydro-4-oxo-7-(1-piperazinyl)-3-quinolinecarboxilic acid] is a broad-spectrum antibiotic belonging to the second-generation fluoroquinolone compounds family (Figure 1). This product is becoming one of the most used antibiotics for human and veterinary clinical applications [11]. Unfortunately, the use of this drug, which is delivered at high doses, has low solubility in physiological conditions [11].

Growing concern regarding the detrimental effects of emerging antibiotic pollutants, such as cipro, on human and animal health needs the search and development of remediation strategies aimed at reducing their presence in soils and aquatic ecosystems [12]. To achieve optimal results, the selection of appropriate absorbents is crucial [9]. 

Clay minerals are layer silicates and natural ubiquitous materials which are often found in soils, rivers, atmospheres, sea floor, and in other moons and planets. They can have high specific surfaces, as well as a large capacity for the adsorption of molecules, water, and organics. They have small particle sizes and layer structures and have confined nanospaces in consequence. They provide selective diffusion properties acting as authentic inorganic membranes. The adsorption of several antibiotics of the cipro family into clay mineral fractions of several soils was reported [13,14,15]. Montmorillonite, a type of clay mineral, exhibits high capacity for adsorbing antibiotics, such as tetracycline, oxytetracycline, and specifically enrofloxacin, which shares structural similarities with cipro [16]. The mechanism underlying this adsorption phenomenon involves the intercalation of these antibiotic molecules into the interlayer space of montmorillonite [16]. This efficacy as an adsorbent can be attributed to its expansive surface area, cation exchange capacity, and swelling properties [9,17]. The quantity of adsorbed cipro depends on the interlayer spacing and adsorption capacity of montmorillonite, displaying higher cipro adsorption than non-swelling clay minerals [16,18]. The investigation of the adsorption behavior and underlying mechanisms of cipro on clay minerals play a pivotal role in elucidating the fate of organic pollutants within the environment [16]. Previous studies have explored the potential mechanisms of cipro adsorption onto minerals, including the formation of surface complexes and cation bridging. However, the primary interaction in the cipro–montmorillonite system still remains uncertain [18].

On the other hand, clay minerals can be used as functional materials for controlled-release drug delivery systems. In the last decades, the application of clay minerals for therapeutic use is extending to many drug families [19,20]. Recently, hybrid hydrogels based on hectorite and halloysite have been prepared with ciprofloxacin as carrier systems for controlled release [21], and wound healing applications [11].

One of the aims of this work is to evaluate the adsorption capacity and interactions of soil samples from the Santa Rosa-Corrientes zone at Argentina for cipro as an antibiotic reference for environmental assessment. In effect, Santa Rosa is a region of Corrientes close to a lagoon with periodical flooding, and a spectacular increase in intensive livestock exploitation, without a regular sewer system and groundwater irrigation. The presence of cipro in human and livestock wastes is one of the main pollution handicaps in this region. The understanding and interpretation of these experimental results are facilitated through atomistic modelling calculations of cipro into the interlayer space of montmorillonite as one representative model of a swelling clay mineral.

## 2. Results and Discussion

### 2.1. Experiment with the Soil Samples

#### 2.1.1. Physical–Chemical Properties

The soil samples are mainly sandy soils with a small amount of clay minerals (Table 1). The soil samples taken at 0–10 cm depth have the lowest proportion of sand with a higher relative proportion of clay than the deeper samples. From the textural analysis, our soil samples have a sandy texture [22]. The textural analysis shows that all the soil samples are sandy and the highest content of sand is 91.65% with respect to clay 4.18%, defining also the percentage of silt. Previous studies identified the presence of montmorillonite in the clay mineral fraction of these soils [23]. This analysis allows us to know the soil fraction that behaves as a colloid, such as clays, which is important for the adsorption–desorption process. The physical–chemical properties of the soil samples show a low soil conductivity (0.117–0.049 dS·m^−1^), which is associated with a low salt content (Table 2). The amount of organic matter is very low in all the samples, and the cationic exchange capacity (CEC) is low, owing to the low clay content. The K^+^ and Na^+^ content increases with the depth of soil samples, whereas the Ca^2+^ and Mg^2+^ content decreases slightly along the soil depth. Nevertheless, this tendency is not linear because the amount of Ca^2+^ and Mg^2+^ increases with the clay content. Hence, the main adsorption capacity comes from the clay minerals component.

#### 2.1.2. Adsorption Isotherms

**Figure 2:**
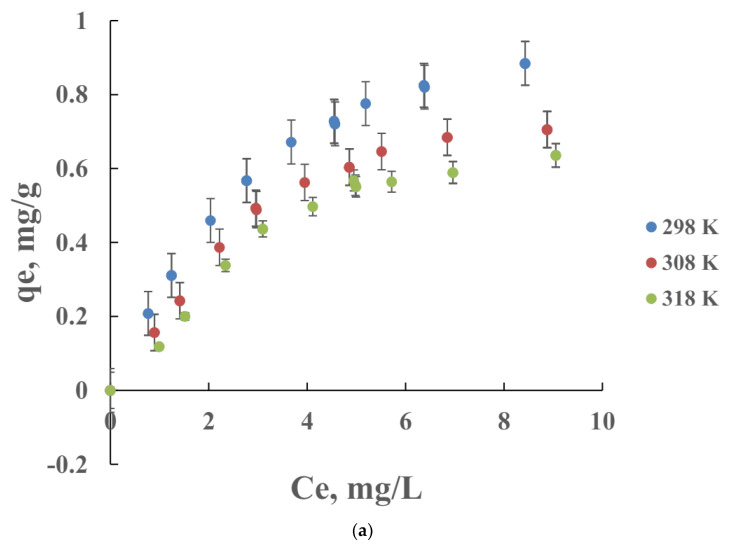

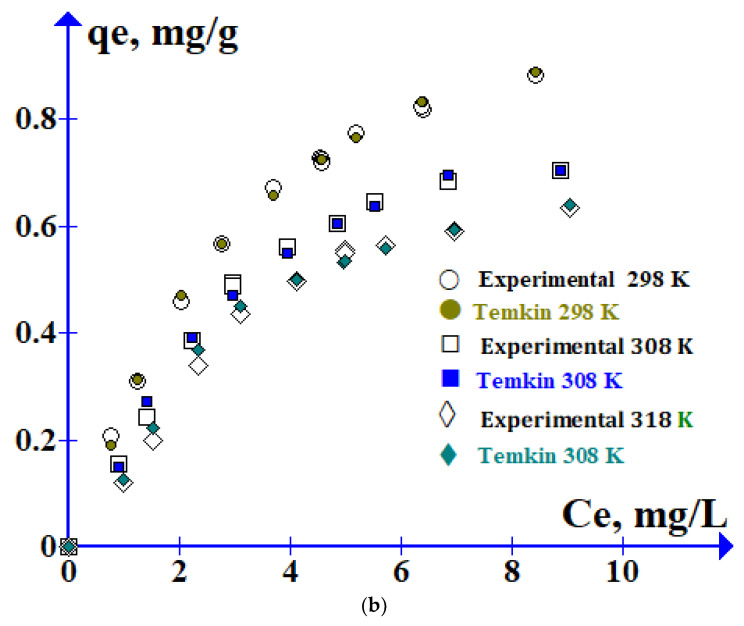
Isotherms of cipro adsorption in soils taken from 1–10 cm depth at several temperatures (**a**) and adjusted to the Temkin model (**b**) studied in this work.

The adsorption isotherms were applied in the sample taken at 1–10 cm depth due to its higher content of clay than other samples (Figure 2a). The adsorption capacity increases with the initial cipro concentration until reaching a plateau. This behavior is consistent with previous works [24,25] and indicates that the driving force is the increasing of concentration. However, at high concentrations, the percentage of adsorbed drug is lower due to the saturation of the active sites of the soil. On the other hand, the percentage of cipro adsorbed by the soil decreases with increasing temperature and may be related to the greater solubility of the drug. 

According to the classification of Giles [26], the isotherms of cipro are type L subtype 3, which suggest physical adsorption and multilayer adsorption that also occurs in adsorption with dilute solutions. The affinity between the drug and the soil is highlighted by the high slope of the initial part of the isotherm. The amount adsorbed increases more slowly when the concentration of the drug in the liquid phase increases. 

#### 2.1.3. Balance Model

Our adsorption process was studied by applying several isothermal models: the Freundlich, Langmuir, Temkin, and Dubinin–Radushkevich models (See Supplementary Materials). Then, our experimental data were fitted by the least square method to these models. The parameters of each isotherm model are presented in Table 3.

The regression approach of determining isothermal parameters appears to offer acceptable fits to the experimental data with good respective regression coefficients (R^2^ values) (Table 3). The statistical indices, RMSE, and R^2^ values suggest that the Temkin isotherm model is the best model for describing the experimental cipro sorption data over the entire range of concentrations and temperatures (Figure 2b). Small discrepancies are detected at low concentrations due to approximations of the model [27]. The Temkin model assumes that the sorption of all molecules in the layer decreases with coverage in all layers of the adsorbent, due to sorbent–sorbate interactions, and that sorption is characterized by a uniform distribution of binding energies, until a maximum binding energy is reached. The parameter K_T_, corresponding to the maximum binding energy of the Temkin model, shows a decrease with increasing temperature, in the same way the value of the parameter β, which indicates the heat of adsorption, decreases and has the lowest value at the highest temperature evaluated of 318 K. These two behaviors suggest the exothermic nature of the adsorption process. The relatively low value of the adsorption energy would indicate that the adsorption process would be physisorption [28,29]. The energy values obtained from the Temkin isotherm are in the range of 2.99–7.51 kJ mol^−1^ < 8 kJ mol^−1^, which indicates a physical adsorption. In the physisorption process, the adsorbates adhere to the adsorbent through weak interactions [30]. 

The fit of the Langmuir model to soils was evidenced in other works [31,32,33,34]. In this model, R_L_ values are less than 1, and around 0.5, indicating a favorable adsorption. However, K_L_ represents the affinity constant that indicates the force with which an adsorbate molecule is retained on the soil surface and in our soil, this Langmuir model does not describe properly our experimental data. In the Freundlich model, the constant K_F_ describes the adsorption force; the higher its value, the stronger the solute adsorption. In all the adsorption processes studied, the value of *n* is greater than 1, which also indicates that they are favorable processes [35]. The isotherms fitted to the Freundlich model show a possible formation of multilayers; however, this model does not fit our experimental data. This model assumes that the surface of the adsorbent is heterogeneous and that the adsorption sites have different affinities; the active adsorption positions with the greatest affinity are filled first and the rest are filled last. This model proposes a complex adsorbate–adsorbent physical union. 

#### 2.1.4. Desorption Isotherms

**Figure 3:**
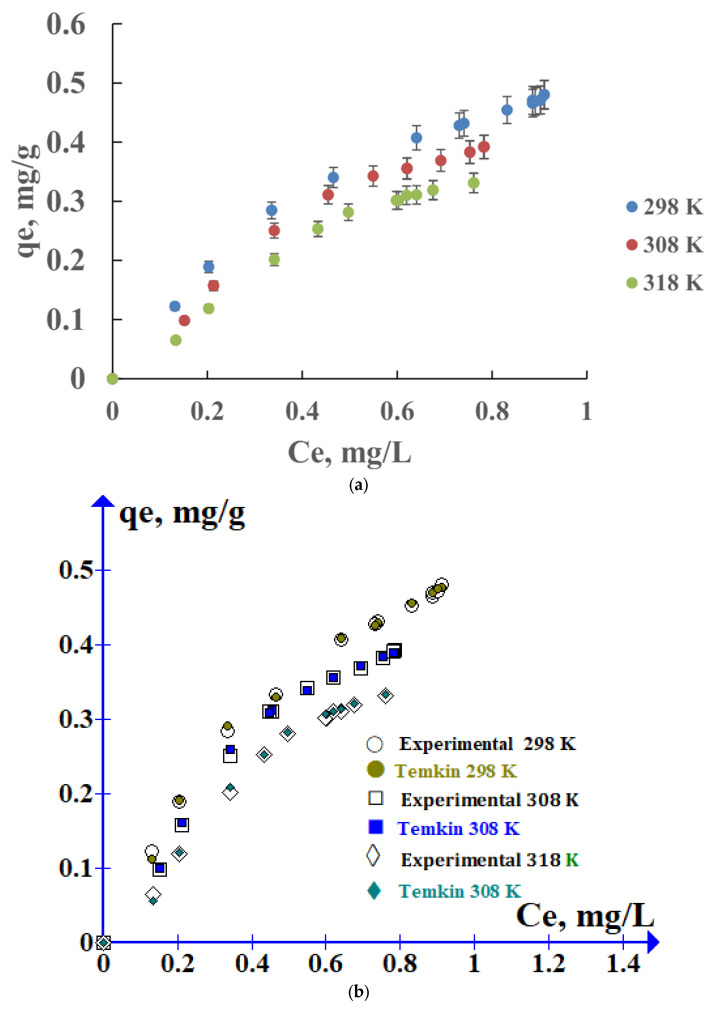
Isotherms of cipro desorption in soils taken from 1–10 cm depth at several temperatures (**a**), and adjusted to the Temkin model (**b**).

Desorption is defined as the passage of the adsorbed solute from the solid phase to the liquid or gas phase. By knowing this process, it will be possible to infer the adsorption force and the possible release of the adsorbed compound by the modification of some environmental factors. The desorption mechanism of the compound will mainly depend on the adsorption energy; the higher this energy, the more difficult it will be for the pollutant to desorb to the soil aqueous media. The desorbed cipro in dissolution increases with the cipro content in the soil (Figure 3a). Nevertheless, no clear tendency to reach a plateau is observed. However, a significant amount of cipro remains in the soil. Up to 60% of the cipro would be retained in the soil (Figure 3a and Figure S1). This fact can indicate that cipro is strongly adsorbed by the soil, with a certain option for passing into the water column.

Our experimental results of cipro desorption isotherms are also correlated with the Freundlich, Langmuir, Temkin, and Dubinin–Radushkevich models (See Supplementary Materials) (Table 4). Again, the Temkin model gives the best fitting with our experimental data at all temperatures studied (Figure 3b), confirming a physical desorption type. As in adsorption, the values of the Temkin parameters of desorption, K_T_, and β decrease with temperature, the process being exothermic in nature.

The desorption mechanism can be studied through the phenomenon of hysteresis, which is defined as the degree of reversibility of the adsorption process [36]. The Langmuir model showed a hysteresis value (H) close to zero, which is considered an irreversible process. However, this model does not fit well to our experimental data. On the other hand, the Freundlich constants obtained for desorption are greater than the K_F_ results for the adsorption processes and consequently, the hysteresis coefficients are less than unified. This can be interpreted that the mobility of cipro through the particles of these soils is slower than the adsorption process, and cipro cannot be removed efficiently from the soil.

Hence, these studies seem to suggest two adsorption mechanisms: one in the confined interlayer space of the clay components of soil with a strong interaction; and a weak additional physical adsorption on, probably, the external surfaces of soil particles. Therefore, only a partial amount of cipro adsorbed physically will desorb in consequence.

#### 2.1.5. Thermodynamic Study

The study of thermodynamic parameters, such as Gibbs free energy (∆G°), adsorption enthalpy (∆H°), and entropy changes (∆S°), allows us to specify the nature of the adsorption process and predict the influence of temperature (Table 3). The enthalpy of this adsorption shows an exothermic process; however, the entropy indicates a complex adsorption process yielding a process with negative free energy. Hence, this indicates that the adsorption process of cipro in this soil is energetically favorable and spontaneous. The values of ΔG° in the adsorption of cipro indicate the tendency to decrease with increasing temperature, demonstrating a greater adsorption capacity of cipro on the soil at low temperatures [37].

The negative ΔH° value obtained for the adsorption suggests an exothermic process. The limits between physical and chemical adsorption are variable. Some authors suggest that the adsorption enthalpy for physisorption is less than 40 kJ/mol [38,39], while others state that the value would be less than 50 kJ/mol [40]. In any case, the adsorption of cipro on soil with a value less than 40 kJ/mol has a physical nature, with the main suggested interactions being electrostatic interactions, hydrogen bonding, and dipole–dipole interactions. Negative values of entropy ΔS° suggest an increase in the order during adsorption due to the confinement of the adsorbates into the adsorbents [41]. 

### 2.2. Molecular Modeling

#### 2.2.1. Cipro Structures

**Figure 4:**
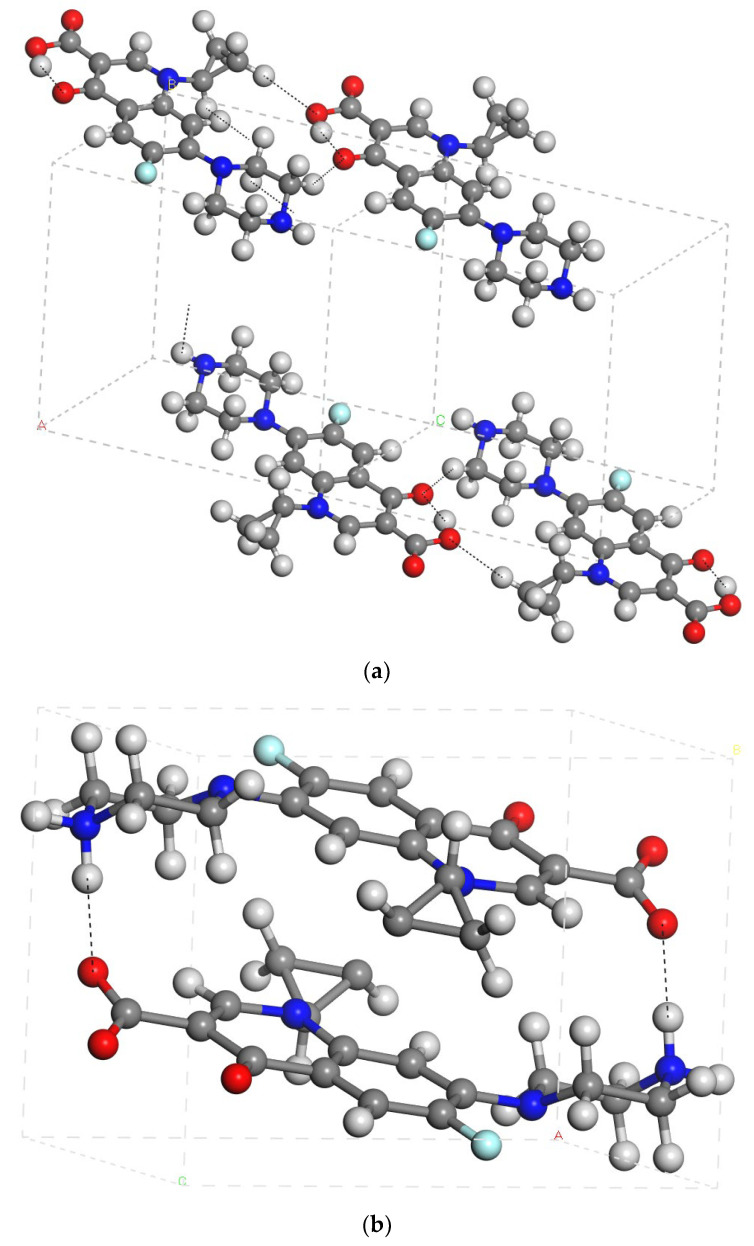
Crystal structure of cipro as a non-ionic (**a**) and zwitterionic (**b**) molecule optimized with INTERFACE and ESP atomic charges.

In order to validate the calculation methodology used in this work, we fully optimized (atomic positions and crystal lattice cell parameters) several crystal structures of cipro with INTERFACE [42] and COMPASS FF [43]. Different net atomic charges were explored: those provided by each FF, those calculated with the QEq method [44] based on the atomic electronegativities, and those calculated by the DFT (Dmol^3^) [45] method associated with the molecular electrostatic field (ESP) [46]. All approximations yielded cell parameters consistent with the experimental values (Tables S1 and S2 in the Supplementary Materials). The crystal cell parameters of cipro as a non-ionic molecule calculated with INTERFACE using the ESP net atomic charges (*a* = 8.08, *b* = 9.85, *c* = 10.42 Å, α = 97.9°, β = 108.8°, γ = 97.1°) are the closest to the experimental ones (*a* = 8.06, *b* = 9.73, *c* = 10.32 Å, α = 99.9°, β = 104.5°, γ = 98.1°) [47] (Table S1 and Figure 4a). The carboxylic H atom forms an intramolecular hydrogen bond with the vicinal carbonyl group d(COH…O=C) = 1.79 Å. A weaker intermolecular hydrogen bond was observed between the N-H groups d(NH…N(H)-C) = 2.29 Å. Additionally, some attractive electrostatic interactions between the carboxylic and carbonyl O atoms with H atoms of vicinal molecules d(C-O…HC) = 2.52–2.57 Å are also relevant. No interaction between the carboxylic and amino groups is observed; however, the aromatic rings are parallel, facilitating the intermolecular π-π interactions stabilizing the packing in the crystal structure. The carboxylic group is coplanar with the aromatic ring and the cyclopropyl group is twisted from the aromatic ring.

In the crystal forms with cipro in the zwitterionic form, the crystal cell parameters calculated with INTERFACE using ESP atomic charges (*a* = 8.32, *b* = 8.38, *c* = 10.87 Å, α = 89.6°, β = 86.2°, γ = 90.5°) closely reproduce the experimental ones (*a* = 7.89, *b* = 8.50, *c* = 10.75 Å, α = 87.4°, β = 84.5°, γ = 88.1°) [48] (Table S2). Therefore, this methodology is validated to be used in the rest of this work. We found that both crystal forms, CCDC2002394 [48] and CCDC714344 [49], are the same crystal structure with the only difference is that the first one was measured at 100 K, whereas the second one was measured at 298 K. In the crystal structure, there is an intermolecular hydrogen bond between the carboxylate O atom and the ammonium H atom (in piperazin ring). In addition, the cyclopropyl ring is twisted with respect to the aromatic ring (Figure 4b).

**Figure 5:**
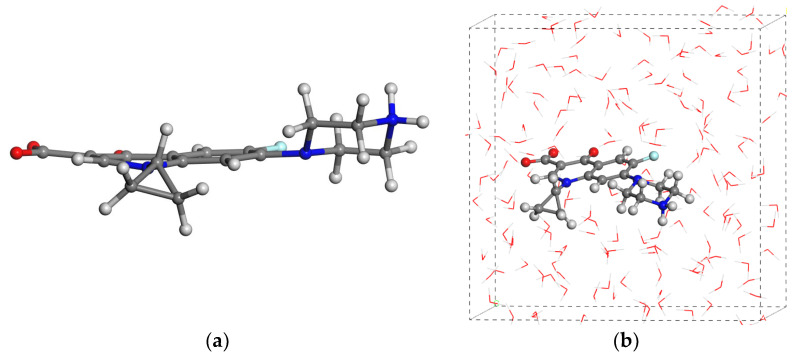
The most stable conformer of the cipro molecule in its zwitterionic form optimized as an isolated molecule (**a**) and solvated (**b**) in a periodical water box (H and O atoms are in white and red).

The cipro molecule was placed as non-ionic form and as a zwitterionic one in the center of the respective empty periodical boxes of 18 × 18 × 18 Å and fully optimized. Comparing the energies of the isolated molecules and the crystal forms, we calculated the packing energy, being −89.85 and −156.05 kcal/mol per cipro molecule for the non-ionic and zwitterionic forms, respectively. Therefore, the zwitterionic form of cipro is more stable than the non-ionic one. This is consistent with previous studies [50]. We applied the DFT calculations for the ESP atomic charges of the cipro molecule optimized with Dmol3, and no charge transfer was observed during the optimization. A conformational analysis was performed scanning small variations of torsional angles and optimizing these conformers for this molecule considering the main dihedral torsion angles of the carboxylate group, the piperazin ring, and the cyclopropyl ring with respect to the aromatic ring. The most stable conformer among 216 conformers was selected for further studies, where the carboxylate group is quasi planar with the aromatic ring (2°), and the piperazin moiety (47°) and cyclopropyl ring (80°) are twisted with respect to the aromatic ring (Figure 5a) 

Cipro molecules as non-ionic and zwitterionic forms were solvated within a water box filled by a Monte Carlo method, and were fully optimized by using the INTERFACE FF with ESP atomic charges as a validated calculation method. The conformations of the molecule remain similar to those of the isolated molecule. The cyclopropyl and alkylheterocycle rings are twisted with respect to the aromatic ring (Figure 5b). The hydration energies of the non-ionic and zwitterionic forms of cipro were −60.25 and −91.13 kcal/mol, respectively. These values indicate that the zwitterionic form exhibits higher solubility compared to the non-ionic form. However, it is important to note that these hydration energies are lower than the packing energies discussed above in both cases. This fact supports the low solubility of cipro crystals in water.

#### 2.2.2. Adsorption of Cipro into Mont

**Figure 6:**
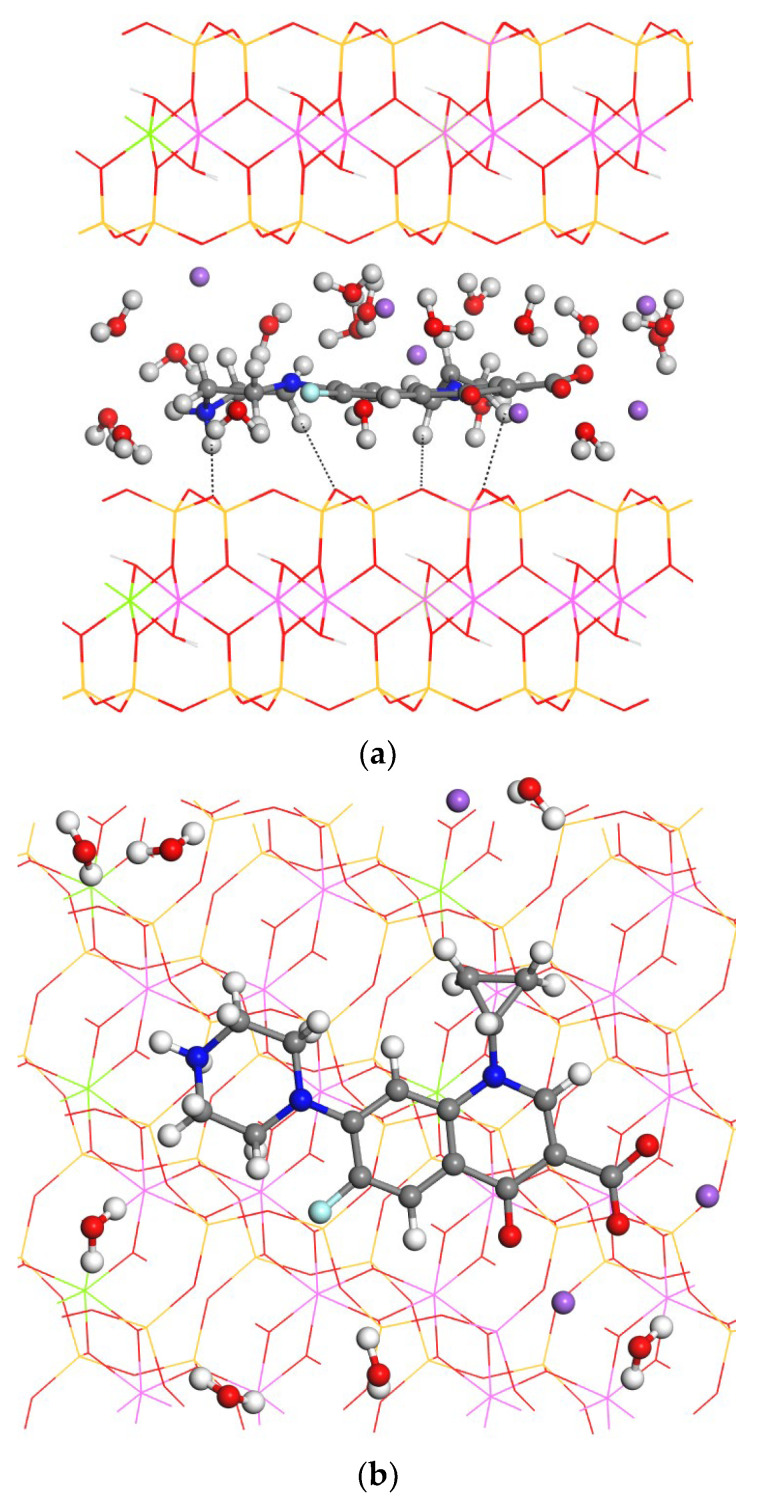
Optimized adsorption complex of cipro and mont, views from (100) (**a**) and (001) (**b**) planes. The H, C, O, F, N, Si, Al, and Na atoms are in white, grey, red, clear-blue, blue, wellow, pink, and purple, respectively. The interlayer atoms are highlighted as balls.

The montmorillonite (mont) structure was optimized with INTERFACE (*a* = 5,16, *b* = 8,94, *c* = 11.7 Å, α = 87.8°, β = 90.6°, γ = 90.1°), whose structure is consistent with the experimental structure (*a* = 5.17, *b* = 8.90, *c* = 12.4 Å, α = 90.0°, β = 99.7°, γ = 90.0°) [51]. One molecule of cipro was placed in the center of the interlayer space of mont (5.4% *w*/*w*). The adsorption of cipro in the interlayer space of the 3 × 2 × 1 supercell of mont was calculated in the same conditions as above. Upon the full optimization (atom positions and lattice cell) of the whole complex, the intercalated cipro molecule adopted a completely different conformation being more linear and quasi-planar in conformation, where the carboxylate group and piperazin moiety are quasi-coplanar with respect to the aromatic ring and the cyclopropyl ring is twisted with the aromatic one. The adsorbate is close to the center of the interlayer space, where the ammonium H atoms are oriented to the O atoms of the mineral surface and the carboxylate O atoms are close to one Na^+^ cation (Figure 6). We notice that no chemical reactive process and charge transfer was studied, due to the physisorption type of the experimental sorption.

Considering the reaction:

Mont + cipro = mont-cipro

The adsorption energy can be calculated as follows:

*E*_ads_ = *E*_mont-cipro_ − *E*_mont_ − *E*_cipro_

Then, the adsorption energies for the non-ionic and zwitterionic forms of cipro into the interlayer space of mont were 32.4 and −18.9 kcal/mol, respectively. This means that the intercalation of the zwitterionic form of cipro is more favorable than the non-ionic form.

In other calculations with INTERFACE, we placed two cipro molecules intercalated in the center of the interlayer space of the 3 × 2 × 1 supercell of mont. The whole complex was optimized with both cipro molecules in the center of the interlayer space of mont. Then, the reaction will be as follows:

mont + 2cipro = mont-2cipro

Then, the adsorption energy of the two cipro molecules can be calculated as follows:

*E*_ads_ = *E*_mont-2cipro_ − *E*_mont_ − 2 × *E*_cipro_

The adsorption energy values were −7.2 kcal/mol for the non-ionic form and −95.4 kcal/mol for the zwitterionic form. These results confirm that the zwitterionic form is more likely to be adsorbed and intercalated in mont than the non-ionic molecule. The presence of water molecules coordinating the Na^+^ cations in mont facilitates the zwitterionic form of cipro. On the other hand, the presence of an additional cipro molecule increases the lipophilia of the interlayer space facilitating the adsorption of the second cipro molecule, being energetically favorable for both cipro molecule forms.

#### 2.2.3. Desorption of Cipro from Mont

When the soil charged with cipro is treated with water media, the cipro molecule is released to the water media during the desorption of cipro. Considering the reaction:

mont-cipro + water = mont + cipro-w

The desorption energy can be calculated as follows:

*E*_des_ = *E*_mont_ + *E*_cipro-w_ − *E*_mont-cipro_ − *E*_water_

where *E*_mont-cipro_ is the energy of the complex with one cipro molecule intercalated in mont, *E*_water_ is the energy of a periodical box with 175 water molecules, *E*_mont_ is the energy of mont, and *E*_cipro-w_ is the energy of the cipro molecule in a box with 175 water molecules. Hence, the desorption energies for the non-ionic and zwitterionic forms of cipro into the interlayer space of mont were calculated with INTERFACE, being −24.6 and −18.7 kcal/mol, respectively. This indicates that the desorption of cipro from the interlayer space of mont to an aqueous dissolution of cipro is energetically favorable.

The soil samples used in the above experiments have a pH close to 5. Hence, a certain proportion of cipro can be protonated (ciproH), and the intercalated ciproH cation replaces one Na cation in the interlayer space of the 3 × 2 × 1 supercell of mont for maintaining neutrality. The desorption of cipro will be by a cation exchange mechanism:

mont-ciproH + NaCl_w = montNa + ciproHCl_w

Then, the desorption energy can be calculated as follows:

*E*_des_ = *E*_mont_ + *E*_ciproHClW_ − *E*_mont-ciproH_ − *E*_NaClW_

where *E*_mont-ciproH_ is the energy of the adsorption complex mont-ciproH with a cipro cation intercalated in mont by cation exchange of the Na^+^ cation, *E_NaCl_* is the energy of a periodical box with solvated Na^+^ and Cl^−^ ions surrounded by 350 water molecules, *E*_mont_ is the energy of mont, and *E*_ciproHClW_ is the energy of the protonated cipro cation with a chloride anion solvated with 350 water molecules into a box. Hence, the desorption energy for the protonated cipro from mont was calculated with INTERFACE, being −14.0 kcal/mol. This indicates that the desorption of the protonated cipro cation from the interlayer space of mont is less energetically favorable than the adsorption of zwitterionic cipro.

Therefore, two mechanisms can occur during the adsorption of cipro in mont: intercalation of one cipro molecule, mainly as the zwitterionic form, per 3 × 2 × 1 supercell of mont; and intercalation of two cipro molecules per 3 × 2 × 1 supercell of mont. Taking into account the low pH of the media, the desorption will be by cation exchange of the protonated cipro molecule being less favorable than the adsorption (Table 5).

### 2.3. Comparison of Experimental and Molecular Modelling Results

Our molecular modelling calculations are consistent with our experimental behavior, where cipro is adsorbed more likely in our soils. Notice that the amount of clay minerals is very low in our soils but it is enough to facilitate the adsorption of cipro. This process is energetically favorable in our calculations mainly when cipro is in the zwitterionic form. Our thermodynamic results obtained from our experimental studies indicate also that this process is exothermic. However, the absolute experimental values do not seem to be quantitatively close to those from molecular modelling energy values. The enthalpy obtained from experiments is −8.60 kcal/mol for our soil (Table 3). This value is consistent with the adsorption energy of our theoretical calculations (−18.9 kcal/mol, Table 5), confirming that cipro will be in the zwitterionic form. The clay fraction is very low (4.18%, Table 1). Additionally, the CEC is also very low of 4.5 mEq/100 g (Table 2). Then, the CEC of the clay mineral fraction will be 4.5/0.0418 = 107.6 mEq/100 g for a monocation like Na^+^ or ciproH^+^; from our models, the CEC will be 107.6 mmoles/100 g. Taking into account that the CEC of montmorillonite can be within the range of 80–120 mmoles/100 g [52], we can consider that the main component of the clay minerals of our soil will be montmorillonite with high CEC and swelling and adsorption properties. This is consistent with previous experimental geological studies of these soils indicating the presence of montmorillonite [23].

Our experiments show that the maximum amount of cipro adsorbed is 0.82 mg/g of solids (Figure 2a, at 298 K). However, 57.3% of the cipro previously adsorbed remains in the solid during the desorption experiments (from Figure 2 at 298 K) being intercalated within the clay mineral. 

Both approaches, experimental and theoretical, follow a similar tendency and the difference in the absolute values can come from the difference in temperature and experimental conditions. At the desorption process, a certain portion of adsorbed cipro is released. The cipro adsorbed on the external surface of minerals is released very easily but the portion intercalated in clay minerals would have more difficulty to be desorbed. 

## 3. Materials and Methodology

### 3.1. Materials

Soil samples were taken from Santa Rosa, province of Corrientes, department of Concepción, Argentina, latitude −28.2667 and longitude −58.1167, located on the riviera of the Laurel Ty lagoon, whose economy is based on the extraction and industrialization of wood, floriculture, and livestock. The soil samples were extracted at different depths: 0–10 cm, 10–20 cm, 20–30 cm, and 30–40 cm. The soil samples were homogenized, dried in air for three days, sieved through a 2 mm diameter mesh, and stored in clean dry glass bottles [53]. Cipro was purchased from Sigma Aldrich (Madrid, Spain).

### 3.2. Adsorption Experiments

The adsorption experiments were performed using the batch equilibrium method. The soil adsorption isotherms were performed by mixing 40 mL of a cipro aqueous solution (background electrolyte: 0.01 mol L^−1^ CaCl_2_) at an initial concentration range of 1–10 mg L^−1^, with 0.1 g of soil in a thermostatic shaker at 100 rpm for 24 h at 298 K, 308 K, and 318 K. Blank samples were prepared simultaneously under the same conditions, which showed a negligible loss of cipro during contact time. All experiments were performed in triplicates and the averaged results were applied.

### 3.3. Desorption Experiments

After adsorption, 40 mL of a 0.01 M CaCl_2_ solution was added to the soil for maintaining the same ionic strength, stirring at different temperatures for 24 h. Subsequently, the mixture was centrifuged for 15 min at 3000 rpm, and the concentration of cipro was determined in the supernatant liquid.

### 3.4. Analytical Methods

The concentration of cipro was determined by spectrophotometric methods with a spectrophotometer (METASH model UV-5100B, Mettler-Toledo, Argentina) at a wavelength of 273 nm. The electrical conductivity (EC) was determined by using a conductivity meter that incorporates temperature correction. A saturation extract was prepared by placing 250 g of soil (fraction < 2 mm, air dried) in a beaker adding enough water for saturation and stirring the sample. After leaving overnight, this sample was filtered and the filtrate was collected. The solid was washed with water until the supernatant came out clean [54].

The organic content of soils was determined by the Walkley and Black’s method, where 0.1 g of soil was treated with 2.5 mL of 1 N potassium dichromate and then 5 mL of concentrated (98%) sulfuric acid, and the excess of dichromate was titrated with 0.5 N ferrous ammonium sulfate [55].

The texture analysis was performed according to the Bouyoucos hydrometer method [56], based on the Stokes’ law that relates the sedimentation velocity to the particle in a fluid with a certain density and viscosity. Distilled water was added to 50 g of soil, 5 mL of sodium oxalate and 5 mL of sodium metasilicate (as a dispersing solution) were also added, and the whole mixture was resting for 15 min. The mixture was dispersed with a high-speed disperser for 5 min and transferred to a 1000 mL test tube and the volume was completed to 1 L. The density of suspension was determined with a densimeter. The readings were taken at 40 s, determining the percentage of sand, and at 120 min, determining the percentage of clay. The percentage of silt was determined by subtracting the amount of clay and sand from the weight of whole soil sample. Subsequently, the Textural Triangle was applied for determining the textural class of the sample, considering the percentages of sand, clay, and silt.

For the determination of the exchangeable cations, a sample of soil (0.3 g) was treated with 10 mL of ammonium acetate at pH 7.0 and stirred for 30 min, then centrifuged at 3500 rpm for 5 min, and the supernatant liquid was collected in 50 mL falcon tubes. This operation was repeated two more times until completing 30 mL of solution [55]. The potassium content of the extract solution was determined using flame photometry. The calcium and magnesium content were determined by the titration of EDTA complexes [55]. The CEC of the soil was determined by washing the solid with 10 mL of 96% ethanol, and afterwards it was centrifuged and the supernatant liquid was recovered. This washing was repeated twice in order to remove the excess of ammonium. This solid was treated with 10 mL of a potassium chloride (10%) solution at pH 2.5, stirring and centrifuging. This process was repeated twice, collecting the supernatants. An aliquot of 5 mL of this supernatant liquid was taken and distilled by stream stripping collecting the distillate over boric acid. The subsequent titration with 0.01 N sulfuric acid determined the CEC of the soil [55].

### 3.5. Data Analysis

The adsorbed amount of cipro by the sandy soil was calculated based on the difference between the initial and final concentration in the aqueous phase, as follows:

(1) $$q_{e}=\frac{\left(C_{i}-C_{e}\right)\times V}{m}$$

where *q_e_* is the adsorption amount in the solid phase (mg)/g, *C_i_* is the initial concentration of adsorbate in the fluid phase (mg L^−1^), *C_e_* is the concentration of adsorbate in the liquid in equilibrium (mg L^−1^), *V* is the volume of the solution (L), and *m* is the weight of the soil sample (g).

The experimental isotherms data were evaluated through four adsorption models: the Langmuir, Dubinin–Radushkevich, Freundlich, and Temkin models. The first two assume monolayer adsorption (homogeneous surface) and the last ones assume multilayer adsorption (heterogeneous surface). 

The Langmuir isotherm is a valid theoretical model for adsorption in a monolayer on a completely homogeneous surface with a finite number of identical and specific adsorption sites and with negligible interactions between molecules. The Dubinin–Radushkevich (D–R) model is a more general model, giving information about porosity and adsorption energy. The Langmuir and Dubinin–Radushkevich isotherm models are mainly applied to strong sorptions with possible chemical-sorption, The Freundlich model (F) is an empirical model, not limited to the formation of a monolayer, but it alludes to the possible formation of adsorption multilayers; the surface of the adsorbent is considered heterogeneous [57]. The Temkin model is characterized by a uniform distribution of binding energy up to a certain maximum; it introduces constants, whose values depend on the initial heat of sorption, and it also assumes a decrease in the heat of sorption with the degree of coating. The Freundlich and Temkin isotherm models are mainly applied to processes with physics-sorption. The description of these models is available in the Supplementary Materials.

Thermodynamic parameters reflect the feasibility and spontaneous nature of the macroscopic process; thus, the free energy, enthalpy, and entropy changes can be estimated using the change of the equilibrium constant with absolute temperature (see Supplementary Materials).

Isotherm adsorption models and thermodynamic parameters have been fitted to the experimental data with the least square method by using the MS Excell (2016) Solver function. The root mean square error (*RMSE*) provides an estimate of how well the predictive model fits the target values:

$$RMSE=\sqrt{\frac{1}{n-1}\sum_{i=1}^{n}{\left(q_{e_{i,exp}}-q_{e_{i,pred}}\right)}^{2}}$$

where *q_i,exp_* are the experimental data and *q_i,pred_* are the predicted values. The smaller the value of *RMSE*, the better the predictive model.

The MS Excell Solver function was used to determine the parameters of the models by using the nonlinear resolution method with generalized reduced gradient until a minimum RMSE value was reached.

## 4. Atomistic Modelling Methodology

The atomic positions of the cipro molecule were obtained from crystallographic data of cipro crystals [47,48,49]. Three crystal forms were considered for calculations, two as zwitterionic structures from Ref. [48] (CCDC ref. 2002394) and from Ref. [49] (CCDC ref. 714344), and another as a non-ionic structure from Ref. [47] (CCDC ref. 757817).

A montmorillonite model was selected as a sample of swelling clay minerals with high adsorption capacity that can be present in our soils and represent one of the main adsorbents of these soils, as reported in previous experimental geological studies of these soils [23]. We do not know the chemical composition of the clay minerals present in these soils. The treatment of soils with CaCl_2_ was for maintaining the ionic strength not for cation exchange. However, the presence of Ca^2+^ and Mg^2+^ in these soils is significant (Table 2), but we do not know which mineral components are associated. Therefore, in the face of these uncertainties, we chose a standard model of montmorillonite with the Na^+^ cation in the interlayer space. Nevertheless, the nature of the interlayer cation is not critical for our theoretical studies of adsorption and our simulations can be compared with our experimental behavior. Then, the chemical composition of the unit cell of our model is Na(Si_7.83_Al_0.17_) (Al_3.17_Mg_0.83_) O_20_(OH)_4_. For the adsorption calculations, a 3 × 2 × 1 supercell was built obtaining a composition of Na_6_(Si_47_Al)(Al_19_Mg_5_)O_120_(OH)_24_. The isomorphous cation substitution with Mg^2+^ in the octahedral sheet was performed considering the previous studies of cation ordering in smectites [58,59]. Two water molecules per Na^+^ cation were placed in the interlayer space solvating the interlayer cations, being twelve water molecules coordinating the Na^+^ cations per each 3 × 2 × 1 supercell.

The INTERFACE force field (FF) [42] was used for energy calculations, applying periodical 3-D boundary conditions. The van der Waals (vdW) interactions were calculated with the Lennard–Jones potential, V(r) = ϵ[(σ/r)^12^ − 2(σ/r)^6^], within this FF. The Forcite code was used for geometry optimizations within the Materials Studio package [60]. This FF has probed to yield excellent performance for similar structures to those of this work, meaning this FF is transferable for organics and mineral systems [42,61,62,63,64]. Nevertheless, the COMPASS FF was also used for comparative studies [43]. The Ewald summation method was used for non-bonding interactions, coulombic, and van der Waals in both FF. In order to obtain alternative net atomic charges for the FF calculations, ESP charges [46] associated with the electrostatic potential were obtained with quantum mechanical calculations based on density functional theory (DFT) by using the Dmol3 code [45] with a generalized gradient approximation (GGA) and the Perdew–Burke–Ernzerhof (PBE) correlation exchange functional [65]. Pseudopotentials with semi-core correction (DSPP) were used. The threshold for the self-consistent field convergence in energy was 10^−6^ Ha. 

The interaction studies were conducted under both dry and hydrated conditions. In both cases, a single cipro molecule was placed at the center of the interlayer space within the montmorillonite model. For establishing the adsorption energies, periodical three-dimensional boxes with an isolated cipro molecule in its different forms, non-ionic one, zwitterionic form, and ciproH^+^-Cl^−^ salt were created, placing them in the center of boxes with enough distances with the vicinal boxes to maintain each isolated structure, 18 × 18 × 18 Å. For the hydrated conditions, similar boxes with and without cipro forms were filled in with water molecules at a density of 1 g/cm^3^ by using a Monte Carlo method and the Interface FF. Each structure was optimized at the same above conditions.

## 5. Conclusions

Our investigations have discovered that the Argentinian soils from Santa Rosa can absorb cipro from wastewaters due to the swelling clay mineral component, in spite of its low content. Atomistic computational modelling has shown that this adsorption can be possible, confirming the experimental results. 

Of the four adsorption isothermal models studied (Freundlich, Langmuir, Temkin, and Dubinin–Radushkevich), the Temkin model fits well with our experimental isotherms, indicating a physisorption and exothermic process. In desorption, the model that best fits the experimental data is also the Temkin model, confirming a physical type desorption. Thermodynamics results also indicate the adsorption process of cipro in this soil is energetically favorable, spontaneous, and exothermic with negative entropy. This process decreases with the increase in temperature, being able to be included in the physisorption range.

Our calculations based on the Interface FF have reproduced the crystal structures of cipro in its non-ionic and zwitterionic molecular forms. Our calculations can describe the intercalation of the zwitterionic form of cipro into the montmorillonite clay mineral, being energetically favorable. At a high concentration of cipro, the intercalation of two cipro molecules per 3 × 2 × 1 supercell of montmorillonite is more energetically favorable, due to the increase in hydrophobicity in the interlayer space of the clay mineral. On the other hand, the desorption of cipro from montmorillonite is less energetically favorable than the adsorption process.

Considering both approaches, experimental and theoretical, we can conclude that the adsorption process consists of two ways: one portion of cipro is intercalated in the swelling clay minerals fraction, like montmorillonite, and the other portion, easily desorbed, is possibly adsorbed on the external surfaces of soil minerals. This explanation justifies the low adsorption energy and the desorption of only a portion of the sorbed cipro. One of the main responsible factors contributing to this retention would be the clay minerals content which has a strong tendency of adsorption according to our calculations shown in this work.

Therefore, ciprofloxacin can be adsorbed by this soil, but a portion of cipro would be desorbed and would diffuse through the soil depth into the water column. Only the possible existence of layers with a high content of swelling clay minerals would retard the diffusion of this pollutant to the environment.

## Figures and Tables

**Table 1:** Structural analysis of soil samples.

Sample Depth (cm)	Sand %	Clay %	Lime %	Texture
0–10	91.65	4.18	4.17	sandy
10–20	93.74	2.08	4.18	sandy
20–30	93.74	4.17	2.09	sandy
30–40	97.91	0.00	2.09	sandy

**Table 2:** Physical–chemical properties of soil samples.

Properties	Sample Depth (cm)			
	0–10	10–20	20–30	30–40
Conductivity (dS·cm^−1^)	0.117	0.049	0.064	0.088
pH	4.89	5.06	4.99	4.98
Organic matter (%)	0.67	0.26	0.25	0.17
K^+^ (mEq/100 g)	0.08	0.12	0.11	0.14
Na^+^ (mEq/100 g)	0.03	0.1	0.06	0.09
Ca^2+^ (mEq/100 g)	0.65	0.45	0.55	0.25
Mg^2+^ (mEq/100 g)	1.75	1.1	1.3	1.15
CEC (mEq/100 g)	4.5	3.90	3.57	3.25

**Table 3:** Isothermal and thermodynamic parameters of the adsorption models fitted to the experimental data. RMSE: root mean square of error.

Freundlich						
T (K)	1/*n*	K_F_ (L g^−1^)		R^2^		RMSE
298	0.649 ± 0.03	0.320 ± 0.20		0.985		0.052
308	0.333 ± 0.18	0.348 ± 0.15		0.963		0.065
318	0.343 ± 0.13	0.307 ± 0.16		0.963		0.042
**Langmuir**						
**T (K)**	**q_max_** **(mg g^−1^)**	**K_L_** **(L mg^−1^)**	**R_L_**	**R^2^**		**RMSE**
298	1.941 ± 0.07	0.077 ± 0.10	0.54	0.982		0.045
308	0.929 ± 0.05	0.363 ± 0.20	0.56	0.997		0.019
318	0.794 ± 0.08	0.428 ± 0.10	0.53	0.990		0.019
**Temkin**						
**T (K)**	**β** **(mg g^−1^)**	**K_T_** **(L mg^−1^)**	**b** **kJmol^−1^/mg g^−1^**	**R^2^**		**RMSE**
298	0.416 ± 0.04	1.080 ± 0.12	2.99	0.998		0.007
308	0.269 ± 0.05	1.049 ± 0.14	6.47	0.993		0.014
318	0.244 ± 0.03	1.032 ± 0.11	7.51	0.992		0.018
**Dubinin–Radushkevich**						
**T (K)**	**q_max_** **(mg g^−1^)**	K_DR_ * 10^4^ **(kJ^−2^ mol^2^)**	**E** **KJmol^−1^**	**R^2^**		**RMSE**
298	0.920 ± 0.08	8.52 ± 0.07	23.70	0.983		0.029
308	0.740 ± 0.04	8.08 ± 0.05	24.72	0.983		0.038
318	0.647 ± 0.05	7.72 ± 0.06	26.24	0.991		0.013
**T (K)**	**ΔG°** **(kJ mol^−1^)**		**ΔH°** **(kJ mol^−1^)**		**ΔS°** **(J mol^−1^ K^−1^)**	
298	−10.40 ± 0.15		−35.83 ± 0.15		−85.21 ± 1.01	
308	−9.59 ± 0.15					
318	−8.73 ± 0.15					

**Table 4:** Isothermal parameters of the desorption models fitted to the experimental data. RMSE: root mean square of errors.

Freundlich					
T (K)	1/n	K_F_ (L g^−1^)	H	R^2^	RMSE
298	0.502 ± 0.04	0.498 ± 0.18	0.150	0.990	0.0110
308	0.413 ± 0.10	0.432 ± 0.09	0.160	0.996	0.0153
318	0.378 ± 0.08	0.371 ± 0.08	0.180	0.969	0.0103
**Langmuir**					
**T (K)**	**q_max_** **(mg g^−1^)**	**K_L_** **(L mg^−1^)**	**R_L_**	**R^2^**	**RMSE**
298	0.846 ± 0.02	1.475 ± 0.20	0.54	0.989	0.0060
308	0.599 ± 0.09	2.376 ± 0.10	0.56	0.997	0.0080
318	0.419 ± 0.04	4.843 ± 0.14	0.53	0.993	0.0050
**Temkin**					
**T (K)**	**β** **(mg g^−1^)**	**K_T_ 10^3^** **(L mg^−1^)**	**b** **kJ mol^−1^/mg g^−1^**	**R^2^**	**RMSE**
298	0.185 ± 0.01	1.094 ± 0.12	13.39	0.998	0.0050
308	0.144 ± 0.09	1.063 ± 0.12	17.78	0.998	0.0026
318	0.112 ± 0.02	1.041 ± 0.15	23.60	0.999	0.0032
**Dubinin–Radushkevich**					
**T (K)**	**q_max_** **(mg g^−1^)**	**K_DR_. 10^8^** **(kJ^−2^ mol^2^)**	**E** **kJ mol^−1^**	**R^2^**	**RMSE**
298	0.630 ± 0.03	8.21 ± 0.022	2467.82	0.985	0.0070
308	0.485 ± 0.04	5.37 ± 0.032	3051.39	0.994	0.0036
318	0.409 ± 0.09	4.81 ± 0.029	3224.12	0.956	0.0044

**Table 5:** Calculated interactions (kcal/mol with INTERFACE) of cipro with mont.

Process	Non-Ionic Cipro	Zwitterionic Cipro	ciproH^+^
Adsorption by intercalation (5.4% of cipro in mont)	32.4	−18.9	
Adsorption by intercalation (10.8% of cipro in mont)	−7.2	−95.4	
Desorption by cation exchange			−14.0
Desorption	−24.6	−18.7	

## Data Availability

The original contributions presented in the study are included in the article/Supplementary Materials, further inquiries can be directed to the corresponding authors.

## Supplementary Materials

The following supporting information can be downloaded at: https://www.mdpi.com/article/10.3390/molecules29081760/s1, Table S1: Experimental and calculated crystal lattice parameters of the crystal form with the cipro molecule as a neutral structure (distances in Å and angles in degrees); Table S2: Experimental and calculated crystal lattice parameters of the crystal form with the zwitterionic cipro molecule (at 100 K) optimized with variable volume (distances in Å and angles in degrees); Figure S1: Profiles of adsorption (blue line)/desorption (red line) isotherms of cipro in soils taken from 1–10 cm depth at 298 K (a), 308 K (b), and 318 K (c).
