# Supplementary material for: Adsorption of Ciprofloxacin on Clay Minerals in Argentinian Santa Rosa-Corrientes Soils
Source: Molecules. 2024 Apr 12;29(8):1760. doi: 10.3390/molecules29081760 (PMC11051898; doi:10.3390/molecules29081760)
Supplement: Supplementary file 1 [file molecules-29-01760-s001.zip › molecules-2896730-supplementary.pdf]

Supplementary Materials

Adsorption of Ciprofloxacin on Clay Minerals in Argentinian Santa Rosa-Corrientes Soils

Nelly L. Jorge [1], María V. Garrafa [1], Jorge M. Romero [1], María J. Jorge [1], Lilian C. Jorge [1], Mario R. Delfino [2], Yumeida V. Meruvia-Rojas [3], Alfonso Hernández-Laguna [3,*] and C. Ignacio Sainz-Díaz [3,*]

[1] Laboratorio de Investigaciones en Tecnología del Medio Ambiente, Área de Química Física, Facultad de Ciencias Exactas y Naturales y Agrimensura, Universidad del Nordeste, Corrientes 3400, Argentina
[2] Instrumental Analysis Laboratory, Facultad de Ciencias Exactas y Naturales y Agrimensura, Universidad del Nordeste, Av. Libertad 5460, Corrientes 3440, Argentina
[3] Instituto Andaluz de Ciencias de la Tierra (CSIC-UGR), Av. de las Palmeras 4, 18100 Armilla, Granada, Spain
* Correspondence: a.h.laguna@csic.es (A.H.-L.); ci.sainz@csic.es (C.I.S.-D.); Tel.: +34-958-526299 (A.H.-L.); +34-958460451 (C.I.S.-D.)

Data Analysis methodology

The experimental results were analysed by fitting to four theoretical models: Freundlich, Langmuir, Temkin, and Dubinin Radushkevich (DR).

The Freundlich model supposes that the adsorption energy varies exponentially, assuming that the sites with the highest affinity for the adsorbate are the first to be occupied and the others are occupied later by using the equation:

$$q_e=K_F \,.\, C_e^{1/n}$$

Where K_F is the equilibrium constant and 1/n is a parameter related to the adsorption intensity.

The Langmuir model is, represented by the equation:

$$q_e=(q_{max}\,\alpha.C_e)/(1+\alpha.C_e)$$

Where the parameter q_{max} (mg.g^{-1}) is a constant that denotes the maximum adsorption capacity, while the constant $\alpha= K_L$ (L mg^{-1}) defines the affinity of the adsorbate for the adsorbent. A characteristic of the Langmuir isotherm is to be able for determining a dimensionless constant called the equilibrium parameter (R_L), which explains the favorability of the adsorption process. It is defined by the following equation:

$$R_L=1/(1+K_L.C_0)$$

where C_0 (mg.L^{-1}) is the initial adsorbate concentration. The R_L can indicate that the shape of the isotherms is unfavorable ($R_L > 1$), linear ($R_L = 1$), favorable ($0 < R_L < 1$) or irreversible ($R_L = 0$).

The Temkin model is expressed in the equation:

$$q_e = \beta \ lnK_T + \ \beta . lnC_e$$

Where q_e is the amount of adsorbate at equilibrium (mg.g^{-1}); C_e is the adsorbate concentration in equilibrium solution (mg.L^{-1}). β is a constant related to the heat of adsorption and is defined by the expression β = RT/b, b being the constant related to the heat of adsorption (J.mol^{-1}), T is the absolute temperature (K), R is the gas constant (8.314 J.mol^{-1}.K^{-1}), and K_T is the Temkin isotherm constant (L.mg^{-1}).

The D-R model is expressed mathematically by the following equation:

$$lnq_e = lnq_{max} - K_{DR}.\varepsilon^2$$

where q_e is the amount of adsorbate adsorbed per weight unit of adsorbent (mg g^{-1}), q_{max} is the maximum adsorption capacity, K_{DR} (mol^2 kJ^{-2}) is the constant to obtain the mean adsorption energy E (kJ.mol^{-1}), and Ɛ is Polanyi's potential. Ɛ and *E* are expressed by the following equations:

$$\varepsilon = R.T.ln\left(1 + \frac{1}{C_e}\right)$$

$$E = \sqrt{\frac{1}{2K_{DR}}}$$

Thermodynamic parameters

The heterogeneous equilibrium can be represented with the apparent equilibrium constant K_d defined by the equation:

$$K_d = \frac{q_e}{C_e}$$

, where q_e is the concentration of the cipro adsorbed on the soil. The change in free energy indicates the degree of spontaneity of the process; more negative values reflect a greater sorption. Its variation can be expressed as a function of the change in enthalpy ΔH° (kJ mol^{-1}) and entropy ΔS° ($J.mol^{-1}K^{-1}$) with temperature:

$$\Delta G^0 = \Delta H^0 - T \cdot \Delta S^0$$

, enthalpy and entropy changes come from the regression line of the apparent equilibrium constant at a function of temperature:

$$\ln K_d = \frac{\Delta S^0}{R} - \frac{\Delta H^0}{R\,T}$$

A negative value of ΔH° reflects that the process is exothermic, while negative values of ΔS° indicate a decrease in randomness at the solution-solid interface during sorption.

Results

Table S1.- Experimental and calculated crystal lattice parameters of the crystal form with the cipro molecule as a neutral structure (distances in Å and angles in degrees).[a]

Parameters	EXP	INTERFACE	INTERFACE_QEq	INTERFACE_ESP	CF	CF_QEq	CF_ESP
a	8.06	7.47	7.61	8.08	8.18	7.72	7.23
b	9.73	10.22	9.80	9.85	9.45	9.99	10.02
c	10.32	10.98	10.85	10.42	10.58	10.42	11.53
α	99.9	102.1	106.0	97.9	105.0	107.0	106.4
β	104.5	103.6	96.1	108.8	97.9	97.1	99.9
γ	98.1	98.4	92.4	97.1	100.4	100.3	107.7

[a] EXP = experimental values; INTERFACE = calculated with the INTERFACE FF with net atomic charges assigned for the own FF, or by the QEq method, or as ESP charges calculated at DFT level; CF = calculated with the COMPASS FF.

Table S2.- Experimental and calculated crystal lattice parameters of the crystal form with the zwitterionic cipro molecule (at 100 K) optimized with variable volume (distances in Å and angles in degrees).[a]

Parameters	EXP	INTERFACE	INTERFACE_QEq	INTERFACE_ESP	CF	CF_QEq	CF_ESP
a	7.89	8.31	8,71	8.32	7.96	8.60	8.12
b	8.50	8.03	8.62	8.38	8.12	8.66	8.33
c	10.75	11.39	10.31	10.87	11.30	10.02	10.63
α	87.4	94.0	88.9	89.6	91.2	88.9	89.8
β	84.5	93.0	82.8	86.2	91,6	81.1	81.6
γ	88.1	89.5	92.1	90.5	90.5	91.7	91.1

[a] EXP = experimental values; INTERFACE = calculated with the INTERFACE FF with net atomic charges assigned for the own FF, or by the QEq method, or as ESP charges calculated at DFT level; CF = calculated with the COMPASS FF.

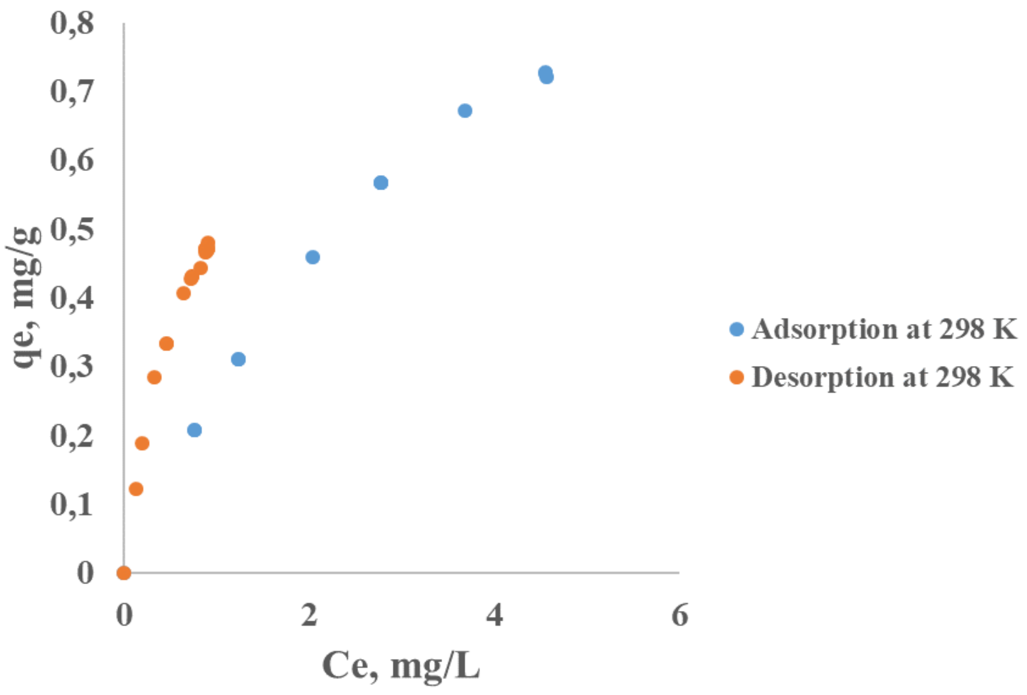

a

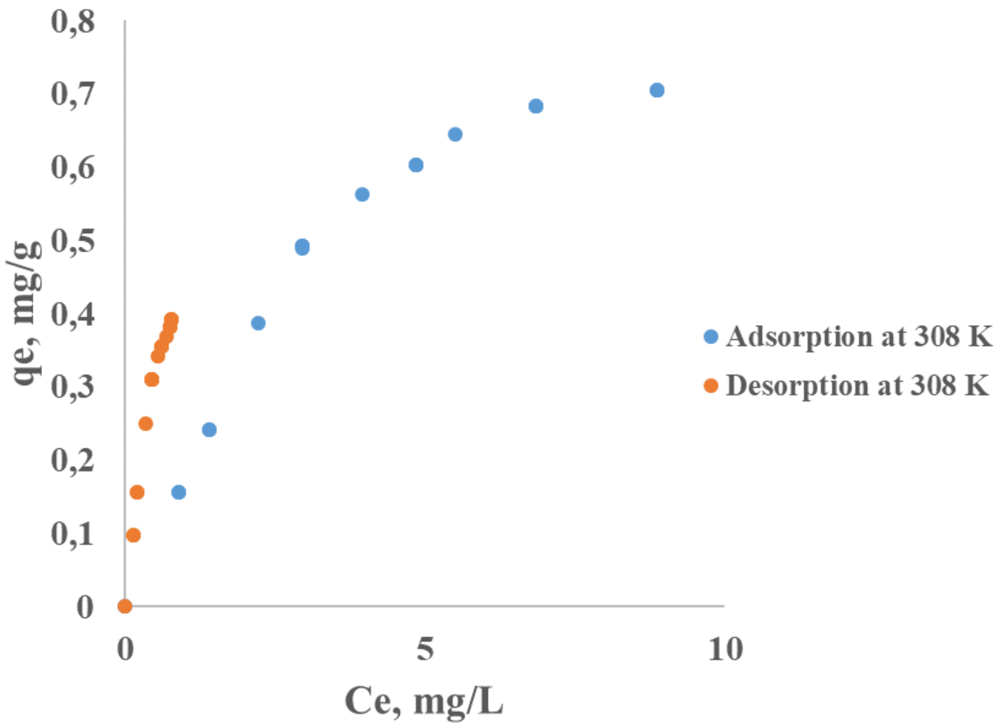

b

(c)

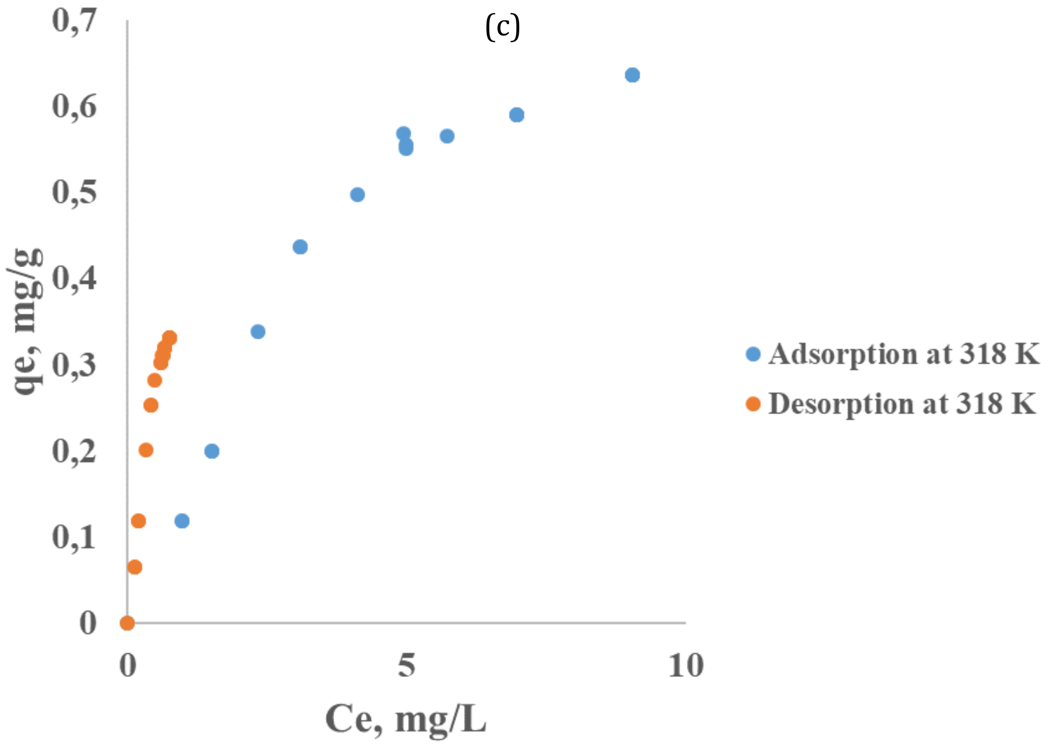

c

Figure S1.- Profiles of adsorption (blue line) / desorption (red line) isotherms of cipro in soils taken from 1-10 cm depth at 298 K (a), 308 K (b), and 318 K (c).
